# Management of hypertension and multiple risk factors to enhance cardiovascular health - a feasibility study in Singapore polyclinics

**DOI:** 10.1186/s12913-016-1491-6

**Published:** 2016-07-08

**Authors:** Tazeen H. Jafar, Ngiap C. Tan, John C. Allen, Shreyasee S. Pradhan, Paul Goh, Saeideh Tavajoh, Fong M. Keng, Jason Chan

**Affiliations:** Program in Health Services & Systems Research, Duke-NUS Medical School, 8 College Road, Singapore, 169857 Singapore; Duke Global Health Institute Durham, Durham, NC 27710 USA; Department of Renal Medicine, Singapore General Hospital, Singapore, 169608 Singapore; SingHealth Polyclinics, Singapore, Singapore; Centre for Quantitative Medicine, Duke-NUS Medical School, 8 College Road, Singapore, 169857 Singapore

**Keywords:** Hypertension, Systolic blood pressure, Healthy lifestyle index, Motivational conversation, Fixed dose combination

## Abstract

**Background:**

High blood pressure (BP) is a leading contributor to cardiovascular mortality globally. There is scarcity of information on effective health systems interventions to lower BP and reduce cardiovascular risk in Southeast Asian countries. We conducted a pilot exploratory trial on 100 adults aged 40 years or older with uncontrolled hypertension to optimize the design for a structured multi-component intervention in primary care clinics for management of hypertension.

**Methods:**

Two clinics were involved, each enrolling 50 participants, with one as the intervention clinic and the other as the control (usual care). The intervention comprised the following four components: 1) an algorithm-driven intervention using a fixed-dose combination (FDC) antihypertensive treatment and lipid lowering medication for high risk individuals, 2) subsidized FDC antihypertensive medication; 3) motivational conversation (MC) for high risk individuals; and 4) telephone follow-ups of all individuals. The process outcomes were intervention fidelity measures. The outcomes of change in parameters of interest were healthy lifestyle index (composite score of body mass index, physical activity, dietary habit, dietary quality and smoking), adherence to antihypertensive medications, and systolic and diastolic BP from baseline to follow-up at 3 months.

**Results:**

Greater than 90 % fidelity was achieved for 3 of the 4 intervention components. Although not designed for conclusive results, the healthy lifestyle score increased by 0.16 (±0.68) with the intervention and decreased by 0.18 (±0.75) with usual care (*p* = 0.02). Adherence to anti-hypertensive medications at follow-up was 95.3 % in the intervention group compared to 83.8 % for usual care (*p* = 0.01). Systolic and diastolic BP decreased in both intervention and control groups, although statistical significance between groups was not achieved. Hypertensive individuals rated all intervention components ‘highly favorable’ on a Likert scale.

**Conclusions:**

Our findings indicate that the proposed, structured multi-component approach for management of hypertension is feasible for implementation in primary care clinics in Singapore, with some changes to the protocol. The observed improvement in the healthy lifestyle index and adherence to anti-hypertensive medications is promising. A large scale, adequately powered trial would be informative to assess intervention effectiveness on BP and cardiovascular risk reduction.

**Trial registration:**

This trial has been registered at ClinicalTrials.gov. ClinicalTrials.gov number NCT02330224. Registered on 28 December 2014.

**Electronic supplementary material:**

The online version of this article (doi:10.1186/s12913-016-1491-6) contains supplementary material, which is available to authorized users.

## Background

Hypertension is the leading attributable risk factor for mortality in the Global Burden of Disease 2010. Over 1 billion people in the world have uncontrolled blood pressure (BP), and this number is expected to increase to 1.5 billion by 2030 [1].

The implications of uncontrolled BP are even greater in Asian populations for whom the relationship between BP and stroke is steep [2]. Successive reports from the National Health Surveys (NHS) have highlighted the consistently elevated burden of hypertension in Singapore. In the 2010 NHS, one in four adults aged 30 years or older suffered from hypertension [3]. About 50 % of individuals with hypertension had uncontrolled BP, and only half of these individuals were on antihypertensive treatment. Immediate health systems interventions are therefore needed.

A systematic review of 72 randomized controlled trials evaluating several health systems strategies to control BP (self-monitoring, organization of care, educational initiatives directed at patients or physicians, nurse- or pharmacist-led care, automated appointment reminders) suggest benefit of organized or structured care comprising multiple strategies, compared to single interventions [4]. More recently, secondary analysis of pre- and post-implementation of structured hypertension management programs in the insured US population, with components including fixed dose combination (FDC) antihypertensive agents and medical assistant visits for measurement of BP, has demonstrated beneficial impact on BP control [5]. However, there is a dearth of empirical trial evidence on strategies to enhance effectiveness of comprehensive care for hypertension and other chronic conditions in Singapore and countries with similar healthcare infrastructure [6].

Although the vast majority of patients seek care from private GPs, the data from the National Health Survey Singapore 2010 indicated that about 45 % of individuals with chronic conditions (hypertension or diabetes) visit the government subsidized polyclinics. We therefore conducted a pilot exploratory trial on 100 adults with uncontrolled hypertension to optimize the design of a structured multi-component interventional study in the primary care clinics for management of hypertension. The intervention consisted of the following four components: 1) an algorithm-driven intervention using fixed-dose combination (FDC) antihypertensive treatment and lipid lowering medication for high risk individuals; 2) subsidized FDC antihypertensive medication; 3) motivational conversation (MC) for high risk individuals; and 4) telephone follow-ups of all individuals with hypertension by a team of physician-supervised nurse practitioners and nurses. The comparator was usual care in the polyclinics.

The overall aim was to assess feasibility of intervention implementation in polyclinics with regards to fidelity of the main interventional components by using the polyclinic workforce and infrastructure to inform the design and scalability of a future full scale cluster-randomized controlled trial (cRCT). We also evaluated the impact of the proposed strategies on change in healthy life style, adherence to antihypertensive medications, and BP levels. In addition, using linear mixed model analysis, a limited evaluation of intervention acceptability was performed.

## Methods

### Study design

The feasibility study was a non-randomized, parallel arm, cluster allocation of two polyclinics: one clinic to the intervention group and one clinic to the usual care (control) group. The trial protocol was approved by SingHealth IRB, and registered at clintrial.gov NCT02330224.

### Inclusion criteria

All individuals aged 40 years or older who were Singapore citizens or Permanent Residents visiting the recruiting polyclinic at least twice during the last one year with a diagnosis of hypertension and uncontrolled BP (systolic BP 140 mmHg and above, or diastolic BP 90 mmHg and above).

### Exclusion criteria

Active systemic illness including fever, hospitalization during prior 4 months, clinically unstable heart failure, advanced kidney disease (estimated CKD-EPI glomerular filtration rate (GFR) < 40 ml/min/1.73 m^2^ or nephrotic range proteinuria (i.e., 3 g/day or more)), known advanced liver disease, pregnancy, or any other major debilitating disease or mental illness that precluded validity of informed consent [7].

### Screening and recruitment

The polyclinics have a system in place where all individuals with hypertension or diabetes receive a “panel” of fasting blood and urine tests at subsidized cost at the time of initial diagnosis and then annually.

Individuals visiting a study polyclinic for their annual panel of laboratory tests marked “hypertension panel” and aged 40 years or older were identified by the laboratory technicians and referred to the dedicated study research coordinator—present at the laboratory—who invited them to be screened for eligibility in the study. All individuals presenting at polyclinics undergo computerized cardiovascular diseases (CVD) risk scoring (using Singapore version of CVD score for men and women) at triage. BP was measured thrice, in the sitting position with arm rested, using a digital device (OMRON HEM-7300). The average of the last two of three BP readings, obtained three minutes apart, was used to confirm eligibility. Those with BP ≥ 140/90 mmHg were considered to have uncontrolled hypertension. Written informed consent was then obtained by the research coordinator. A total of 100 participants were recruited: 50 in each clinic.

The research coordinator administered a brief study questionnaire for information on socio-demographics. In addition, EQ-5D-5 L was administered to ascertain health related quality of life. Anthropometric measurements obtained were waist, height and weight. All participants were offered light refreshments after completion of the study questionnaire and received a S$5 voucher as reimbursement in both polyclinics.

### Intervention

1. *Training Physicians in Treatment algorithm*: All physicians from the intervention polyclinic were invited for training in the treatment algorithm (Fig. 1). Intensive training sessions (3 h sessions over 2 days) were scheduled during regular continued medical education (CME) hours for the convenience of providers. After CVD risk assessment at triage and measurement of BP by research coordinators, participants were triaged to physicians for evaluation, including prescription of antihypertensive medications per treatment algorithm.

**Fig. 1:**
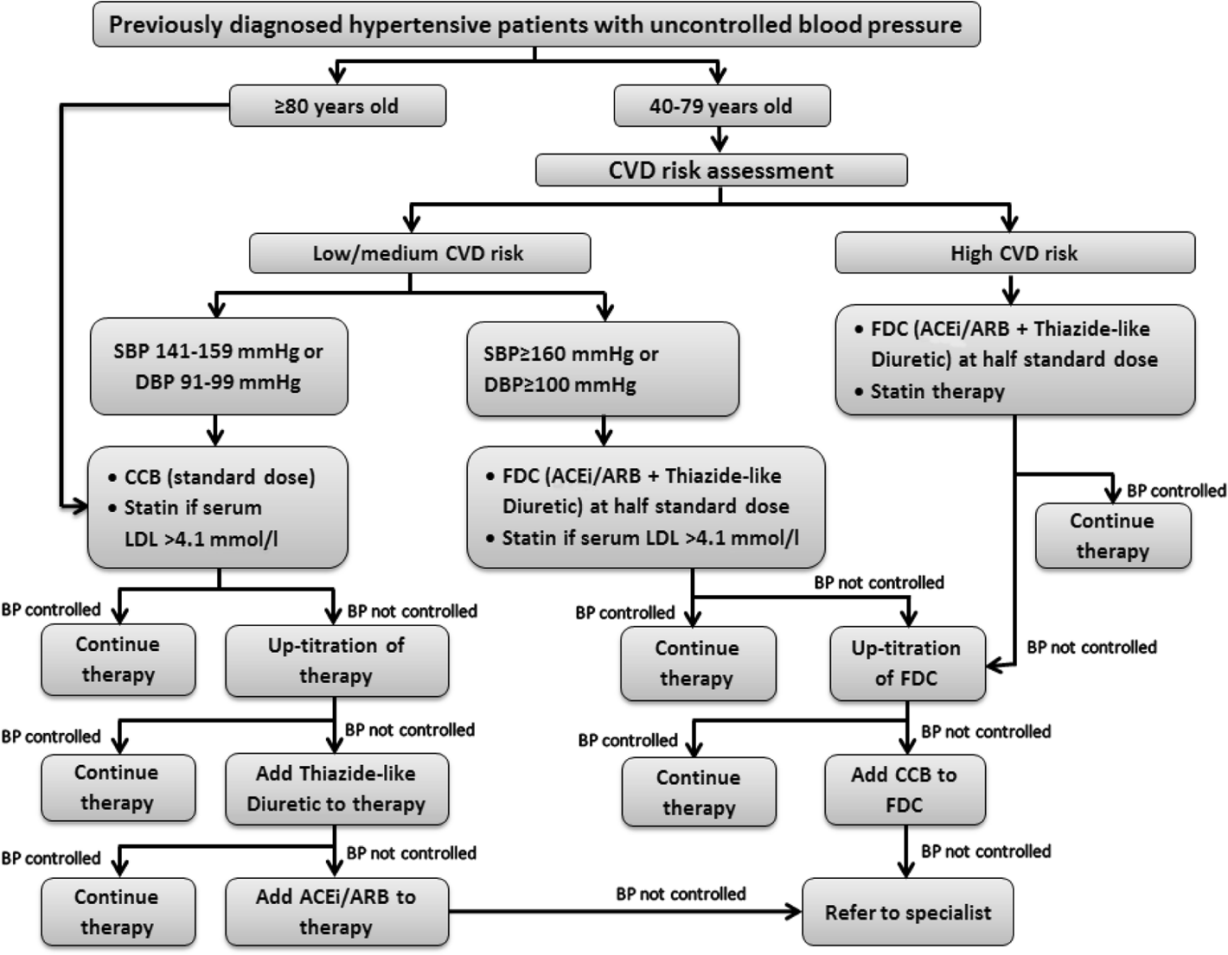
Treatment algorithm
  All participants with a CVD score indicating risk of acute coronary heart disease of 20 % or greater over 10 years, or with diabetes, or pre-existing CVD were categorized as “high risk”. All other participants were categorized as “low/medium risk”. In all “high risk” participants, the angiotensin receptor blocker (ARB), losartan, plus hydrochlorothiazide (HCTZ) diuretic was initiated as a single pill fixed-dose combination (FDC) at half-standard dose of each. Participants were titrated to full dose if BP remained uncontrolled at a subsequent visit in 4–6 weeks and initiated on lipid lowering therapy with statins [8, 9]. This combination regimen has been shown to be effective and safe in lowering BP and preventing CVD in some trials [10–13]. The first-line antihypertensive medication for participants at “low/medium risk” was calcium channel blockers (CCB). The next agents would be ACE inhibitors (ACEI) (preferably in those less than 55 years) or thiazide-type diuretics (preferably in those aged 55 years and older) initiated at full standard dose and up-titrated as necessary [14]. However if BP was greater than 20/10 mmHg above target at the initial visit, FDC therapy was initiated except in the very elderly (aged 80 years or older) in whom therapy with single agent was preferred [15]. The target BP for individuals aged < 80 years was < 140/90 mmHg, and < 150/90 mmHg for the very elderly.
2. *Subsidy to FDC Antihypertensive Medication*: The ARB/HCTZ diuretic FDC antihypertensive medication was subsidized at 50 % of the standard price at the intervention clinic. This subsidy was in line with the existing practice at the government sector hospitals in Singapore.
3. *Training Nurses in Motivational Conversation*: A special curriculum for hypertension management was prepared by the psychologists in consultation with the physicians using relevant case studies. Nurses and nurse practitioners in the intervention clinic were trained for 2 days (4 h session each) by the psychologist. The counseling approach of MC to high risk participants focused on principles of empathy and aimed to strengthen personal motivation and commitment to setting priorities for self-care and medication adherence by eliciting and exploring a person’s own reasons for change within an environment of acceptance and compassion [16]. The duration of a session was recorded.
4. *Structured Follow-up over Telephone*: All participants in the intervention clinic were followed via telephone by the nurses trained in MC, and received advice for adherence to treatment by the nurse at week 4 and week 8 from the time of recruitment. Information on adverse events was also obtained and an action plan (discontinue the suspected drug and arrange visit to clinic, as appropriate) was communicated accordingly. A standardized checklist with questions on lifestyle modification and adherence to medications was used to communicate the key messages (Additional file 1). The average duration of a call was recorded. The nurses discussed the checklists with the physicians on weekly basis.

### Usual care

The health providers in the polyclinic allocated to usual care continued their existing practices. Also, the participants continued to pay for the services (i.e., physician or nurse consultation, any diagnostics or medications) as per their existing model of reimbursement.

### Outcomes assessment

All participants were assessed at the clinic by dedicated research coordinators at baseline and after three months when BP was measured. The research coordinators also called the participants by telephone for outcomes assessment at 6 weeks after baseline and administered a follow-up questionnaire on lifestyle (tobacco use), information on BP self-monitoring, self-care, medication use (traditional medicines) and medication adherence. At the final clinic visit in the intervention group a research coordinator also administered a semi-qualitative questionnaire along with the follow-up questionnaire to all the participants. This semi-qualitative questionnaire captured participants’ perspectives about the telephone follow-up calls and also about the MC and subsidized FDC to those identified as high CVD risk individuals. At the end of the study nurses were interviewed to understand their perspectives on the telephone follow-up calls and MC.

Drug information was extracted through pharmacy dispensing records. During the monthly outcomes assessments, information on these costs and any hospitalizations were also obtained. Reasons for hospitalizations and costs incurred were recorded.

Each participant was given a S$20 grocery store voucher per study visit as remuneration for participation in the study.

### Statistical analysis

The main process outcome of interest was *intervention fidelity* defined as the proportion of a) the planned orientation sessions delivered to physicians and nurses, b) the prescription of FDC to eligible participants, c) the delivery of MC to eligible participants, and d) the telephone follow-ups.

The other key outcomes were differences between treatment groups in change from baseline to final follow-up at three months for the following:

1. Change in healthy lifestyle index (HLI) [17–20] (Additional file 2)
2. Adherence to antihypertensive medications.
3. Change in systolic BP (SBP)
4. Change in diastolic BP (DBP)
5. Quality of life assessed via EQ-5D-5 L

The Healthy lifestyle index (HLI) is a composite score of five variables, namely BMI, self-reported physical activity, dietary habits, dietary quality and smoking (Additional file 2). Score of 0 was considered as poor health while score of 5 was considered to be excellent health [18–20]. Medication adherence was computed using the proportion of days covered (PDC), where length of follow-up was the denominator and total number of days’ supply of medication dispensed was the numerator [21]. In addition, self-reported adherence was also analyzed via Morisky Medication Adherence Scale (MMAS-8) on a continuous scale.

All enrolled participants were included in the analyses on an intention-to-treat basis. Throughout, *p*-values < 0.05 were considered statistically significant. We compared baseline characteristics between the two groups using a 2-sample *t*-test for continuous variables and Fisher’s exact test for categorical variables. The last observation carried forward (LOCF) method was used to impute missing values.

Change from baseline in systolic BP was compared between the two groups using a general linear mixed model with difference in SBP as the dependent variable and independent variables clinic, baseline SBP, age, gender, diabetes and house ownership. The EQ-5D-5 L index score was calculated using the Japan value sets.

The weekly per individual cost of antihypertensive medications in both groups was computed. We also performed a limited tabulation of direct intervention cost of delivering motivational conversation by the nurses, telephone follow-ups, and FDC subsidy at the hypertensive individual level.

Formal sample size calculation was not performed for the feasibility study.

## Results

### Participant flow

**Fig. 2:**
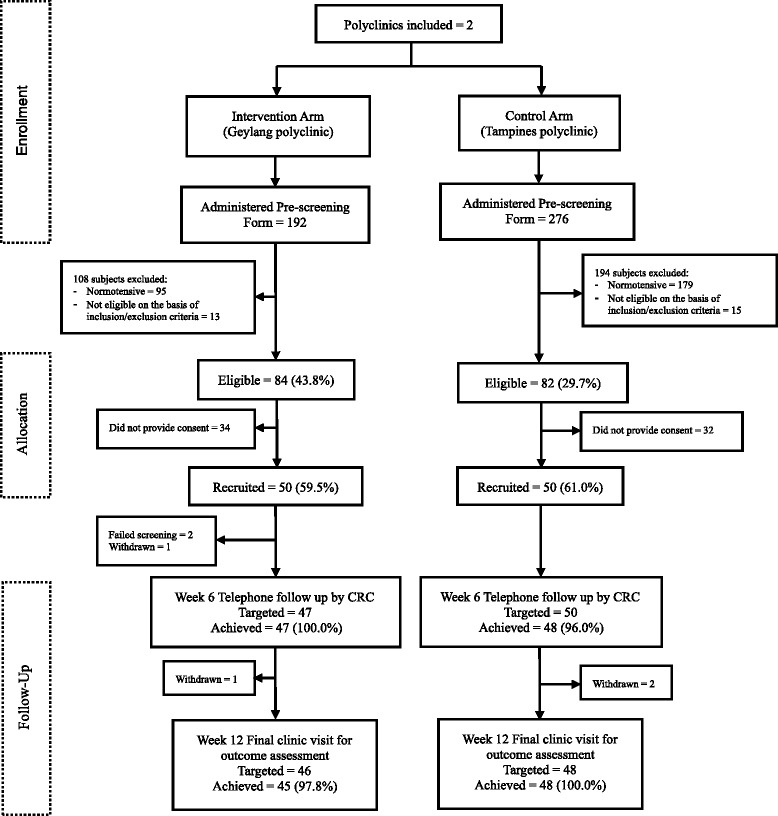
CONSORT flow diagram

A detailed description of recruitment (CONSORT flow diagram) is shown in Fig. 2. The week 6 telephone call follow-up rate by research coordinators was 100 % in the intervention clinic compared to 96 % for usual care. The response rate for the final clinic visit was 97.8 % in the intervention clinic compared to 100 % for usual care in targeted participants.

### Baseline characteristics

As shown in Table 1, participants in the intervention group were older (*p* < 0.001), less educated (*p* < 0.001), greater Chinese ethnicity (*p* < 0.001), and less likely to own a home (*p* = 0.04) than those in the usual care group. The mean age (±SD) in the intervention group was 66 ± 10.3 while that in usual care group was 58.48 ± 8.3 (*p* < 0.001).

**Table 1:** Baseline characteristics of participants by study groups

Characteristic	Intervention	No Intervention	*p*-value^a^
Total (*n* = 100)	(*n* = 50)	(*n* = 50)	
Mean age, y (SD)	66 (10.3)	58.48 (8.3)	<0.001*
Median age, y (IQR)	68 (60.3, 72.0)	59 (52.3, 64.0)	<0.001*
Age (y), *n* (%)			<0.01*
40-59	12 (24.0)	26 (52.0)	
60-74	28 (56.0)	22 (44.0)	
≥ 75	10 (20.0)	2 (4.0)	
Men, *n* (%)	22 (44.0)	25 (50.0)	0.69
Ethnicity, *n* (%)			<0.001*
Chinese	47 (94.0)	23 (46.0)	
Malay	2 (4.0)	16 (32.0)	
Indian	1 (2.0)	10 (20.0)	
Mixed ethnicity	0 (0.0)	0 (0.0)	
Other	0 (0.0)	1 (2.0)	
Religion, *n* (%)			<0.001*
No religion	6 (12.0)	0 (0.0)	
Buddhism	31 (62.0)	6 (12.0)	
Taoism	5 (10.0)	2 (4.0)	
Christian	4 (8.0)	12 (24.0)	
Muslim	3 (6.0)	20 (40.0)	
Hindu	0 (0.0)	4 (8.0)	
Other	1 (2.0)	6 (12.0)	
Education level, *n* (%)			<0.001*
Less than Primary	31 (62.0)	6 (12.0)	
Greater than Primary and less than Secondary	3 (6.0)	0 (0.0)	
Secondary	11 (22.0)	29 (58.0)	
More than Secondary	5 (10.0)	15 (30.0)	
House, *n* (%)			0.04*
Owner	39 (78.0)	47 (94.0)	
Live on rent/Other	11 (10.0)	3 (2.0)	
Heart Disease, *n* (%)	2 (4.0)	2 (4.0)	1.00
Diabetes, *n* (%)	6 (12.0)	12 (24.0)	0.19
Stroke, *n* (%)	3 (6.0)	1 (2.0)	0.62

*Statistically significant

^a^Group difference at baseline using *t*-test for independent groups for continuous variables and Fisher’s exact test for categorical variables (e.g., ethnicity, religion, etc.)

### Intervention fidelity

**Fig. 3:**
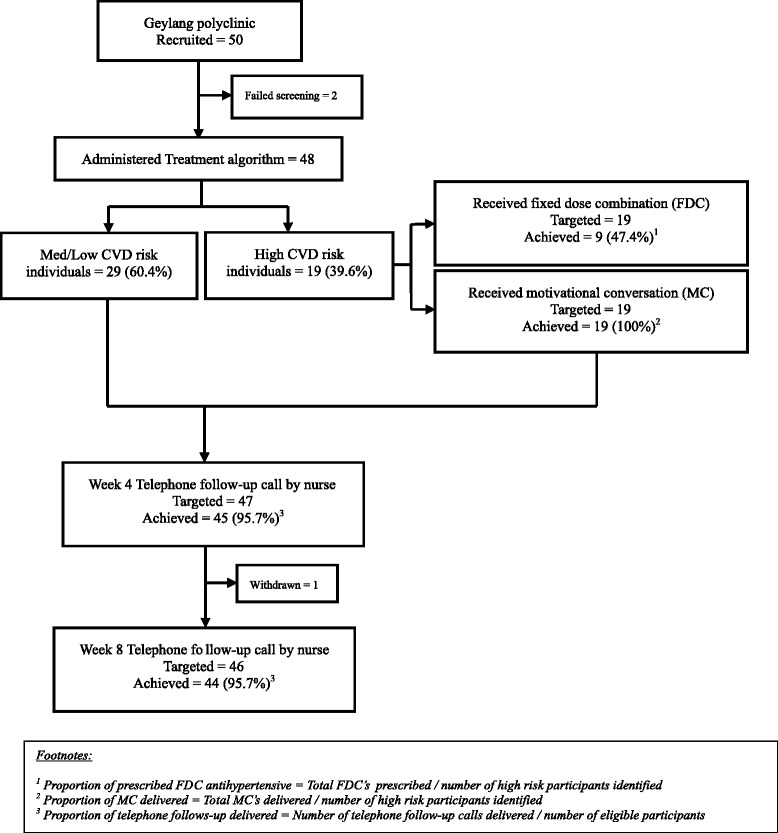
Intervention fidelity

Figure 3 shows intervention fidelity achieved.

#### Training sessions

All (100 %) of the planned orientation sessions on treatment algorithm and MC, including telephone follow-ups were delivered to the participating physicians and nurses. The duration of each physician orientation session was 2 h and each MC session 4 h. Attendance at sessions was over 90 % of expected participants.

#### Prescription of FDC to eligible participants

Amongst those identified as high CVD risk individuals, 47.4 % received a fixed dose combination of antihypertensive medication at 50 % subsidized cost.

#### Delivery of MC to eligible participants

One hundred percent of eligible participants received MC. The average duration of the MC was around 40 min.

#### Delivery of telephone follow-ups

The response rate for week 4 and week 8 telephone follow-up calls by nurses was 95.7 %. The average duration of each call was 14 min.

### Outcomes

During 3-month follow-up, the healthy lifestyle index increased by an average of 0.16 (±0.68) units in the intervention group and decreased by 0.18 (±0.75) in the usual care group, with the difference statistically significant (*p* = 0.02). The adherence to antihypertensive medications was significantly better in the intervention compared to usual care group, both for any antihypertensive medication (*p* < 0.01) as well as all hypertensive medications (*p* = 0.03) (Table 2).

**Table 2:** Change in outcome variables from baseline

Variables	Intervention group	Within-group *p*-value	No Intervention group	Within-group *p*-value	Between-group *p*-value
	(*n* = 50)		(*n* = 50)		
Healthy Lifestyle Index, mean (SD)^a^					
Baseline	2.1 (0.99)		2.4 (1.03)		
Final	2.3 (1.04)	0.43	2.2 (0.89)	0.35	
Change	0.16 (0.68)		−0.18 (0.75)		0.02*
Healthy Lifestyle Index, median (IQR)^a^					
Baseline	2 (1, 3)		2 (2, 3)		
Final	2 (2, 3)	0.54	2 (2, 3)	0.48	
Change	0 (0, 0)		0 (−1, 0)		0.01*
Anti-hypertensive medication adherence					
PDC (any medication class)^b,c^, %	95.27		83.78		<0.01*
PDC (all medication classes)^b,c^, %	92.05		84.47		0.03*
Mean Systolic blood pressure (> = 140 mmHg), mean (SD)					
Baseline	158.0 (11.9)	<0.001*	160.66 (15.7)	<0.001*	
Final^d^	142.79 (13.3)		140.17 (12.9)		
Adjusted change mm Hg, mean (95%CI)^e^	−17.58 (−24.06 to −11.11)		−18.99 (−25.88 to −12.09)		0.58
Mean Diastolic blood pressure (> = 90 mmHg), mean (SD)					
Baseline	94.6 (5.7)	<0.001*	96.86 (11.5)	<0.001*	
Final^d^	80.82 (11.7)		86.86 (10.5)		
Adjusted change mm Hg, mean (95%CI)^e^	−15.6 (−20.85 to −10.35)		−12.93 (−18.48 to −7.37)		0.19

*PDC* proportion of days covered

*Statistically significant

^a^Healthy Lifestyle Index is a cumulative score of 5 variables; BMI, Physical activity, Dietary habit, Dietary quality and Smoking (Additional file 2)

^b^PDC = (Total number of days’ supply of medication dispensed/length of corresponding follow-up) x 100

Ref. Mazzaglia G, Ambrosioni E, Alacqua M, Filippi A, Sessa E, Immordino V, Borghi C, Brignoli O, Caputi AP, Cricelli C, Mantovani LG. Adherence to antihypertensive medications and cardiovascular morbidity among newly diagnosed hypertensive patients. *Circulation*. 2009;120:1598–1605

^c^Adjusted for age and gender

^d^Missing data was imputed by using the mean values of final

^e^Adjusted for age, gender, house ownership, diabetes and baseline values

Mean systolic and diastolic BP declined significantly in both intervention and usual care groups, however, the difference in these changes was not statistically significant. The unadjusted intra-cluster correlations (ICC) for differences in SBP and DBP were 0.02 and 0.33, respectively.

The adjusted quality of life score tended to improve in the intervention group (mean, 0.01; range, −0.04 to 0.06) and decline in the usual care group (mean, −0.02; range, −0.07 to 0.03) (Table 3). The difference in change between groups was not statistically significant (*p* = 0.19).

**Table 3:** Change in outcome variables from baseline

	Intervention group	Within-group *p*-value	No Intervention group	Within-group *p*-value	Between- group *p*-value
	(*n* = 50)		(*n* = 50)		
EQ-5D-5 L, mean (SD)^a,b^					
Baseline	0.94 (0.1)		0.91 (0.1)		
Final	0.96 (0.1)	0.20	0.93 (0.1)	0.28	
Adjusted change, mean (95%CI)^c^	0.01 (−0.04 to 0.06)		−0.02 (−0.07 to 0.03)		0.19
Met-min/week, median (IQR)^d,e^					
Baseline	713 (903.8)	0.09	1125 (1928.3)	0.72	
Final	1253 (693)		1074 (1908.4)		
Change, median (IQR)	0 (603.8)		53.25 (1062.4)		0.52
BMI, mean (SD)					
Baseline	25.75 (4.0)		27.17 (4.6)		
Final^f^	25.64 (3.8)	0.04*	27.50 (4.8)	0.30	
Adjusted change, mean (95%CI)^g^	−1.06 (−2.11 to −0.01)		−0.58 (−1.69 to 0.53)		0.23

*BMI* body mass index

*Statistically significant

^a^EQ-5D-5 L calculated on Japanese version

^b^Missing data was imputed by using the baseline values

^c^Adjusted for age, gender, house ownership, diabetes and baseline values

^d^Met-min/week = (Walk Mets x min x days) + (Mod Mets x min x days) + (Vig Mets x min x days)

^e^Missing data was imputed by using the median value of final

^f^Missing data was imputed by using the mean values of final

^g^Adjusted for age, gender, house ownership, diabetes and baseline values

The nurses who conducted the telephone based follow-ups gave positive feedback in a group session but suggested increasing the interval between the follow-up calls. 86 % of participants were “happy or very happy” with the telephone based follow-up, and 89 % of those receiving MC responded similarly (Table 4).

**Table 4:** Quality assessment

Response	Telephone follow-ups	Motivational conversation
	Target = 45; Achieved = 43 (95.5 %)	Target = 19; Achieved = 18 (94.7 %)
Very happy	12	7
Happy	25	9
Neutral	6	2

The weekly cost of antihypertensive medications incurred by the hypertensive individuals in the intervention and control groups was S$1.80 and S$1.70, respectively (Table 5).

**Table 5:** Consumption and cost of antihypertensive medications

Measures	Intervention group (*n* = 42)	No Intervention group (*n* = 49)
Antihypertensive medications consumed per day (*n*)	1.7	1.4
Cost per week (SGD)	1.8	1.7

The cost of face to face MC was S$12.1 per high risk individual per session (average duration 40 min), monthly telephone follow up call was S$4.5 per individual per call (average duration 14 min), and FDC antihypertensive medication subsidy was S$ 1.4 per high risk individual per week (Table 6).

**Table 6:** ^a^Cost of intervention components

MOTIVATIONAL CONVERSATION	
Mean (SD) time in minutes for delivering one face to face MC session per high risk hypertensive individual	40 (21.5)
^b^Cost of delivering face to face MC per high risk hypertensive individual in Singapore $	12.1 (8.9 to 17.1)^c^
TELEPHONE BASED FOLLOW-UP^d^	
^d^Mean (SD) time in minutes for telephone call by nurses per hypertensive individual	14 (18.7)
^e^Cost of telephone call by nurses per hypertensive individual in Singapore $	4.5 (3.4 to 6.2)^c^
SUBSIDY ON FDC	
Subsidy on fixed dose combination antihypertensive medication per high risk individual per week in Singapore $	1.4

^a^The computation accounts for direct costs only

^b^Average salary of midlevel nurses in the public sector clinic is used in the computation

^c^The parentheses represent the cost from junior nurse to senior nurse

^d^Number of hypertensive individuals who received telephone follow-up call after week 4 and week 8 were 45 and 40, respectively

^e^The cost of telephone call includes cost of nurses’ time plus telephonic charges

## Discussion

Our study on 100 participants with uncontrolled hypertension in 2 polyclinics in Singapore demonstrated feasibility of implementing a structured multicomponent intervention consisting of 1) an algorithm-driven antihypertensive regimen incorporating a fixed dose combination (FDC) antihypertensive and lipid lowering treatment for high risk individuals; 2) subsidy on FDC antihypertensive medications; 3) motivational conversation (MC) for high risk individuals; and 4) telephone follow-ups of all individuals with hypertension with high intervention (> 90 % for 3 of 4 process outcomes). Although not designed to yield conclusive results, the intervention increased the healthy lifestyle score by an average (SD) of 0.16 (±0.68) at the intervention group with a decrease of 0.18 (±0.75) for usual care (*p* = 0.02) over 3 months. The adherence to anti-hypertensive medications at follow-up was 95.3 % in the intervention group compared to 83.8 % in usual care (*p* = 0.01). Systolic and diastolic BP decreased in both intervention and control groups, however the difference was not statistically significant. Participants rated the intervention components (MC, telephone follow-ups, subsidy on FDC) ‘highly favorable’ on a Likert scale. A large scale, adequately powered cluster RCT of the proposed intervention with some additional measures to enhance physician prescription of FDC antihypertensive medications to eligible participants would be informative regarding the effectiveness and cost-effectiveness of the packaged approach for reduction of BP and cardiovascular risk in this population.

The conduct of developmental and exploratory research is important prior to undertaking full-scale trials to evaluate complex interventions or pragmatic trial. Such efforts allow opportunities to modify the approach to be evaluated in a full scale more resource intensive trial. Our feasibility study was conducted from a perspective of introducing practical strategies in local primary care clinics with heavy caseloads. The high fidelity of MC and telephone follow ups were key process outcomes observed in our trial, and the positive feedback from providers and hypertensive individuals indicates that the existing polyclinic infrastructure and workflow can be reconfigured, and the strategies institutionalized for long term delivery of the proposed strategies in the public health infrastructure in Singapore. At the same time, only 50 % of physicians prescribed FDC to eligible participants. Therefore, the full scale study design would need additional measures to further enhance prescription rates. A performance indicator such as a physician management checklist embedded in the electronic health system indicating whether FDC was considered could be an effective option. These strategies would be applicable to many other countries where physicians and non-physician health professionals work as a team for synergistic benefit to achieve BP control, which remains a global public health challenge.

Our feasibility results suggest a potentially favorable effect of the packaged intervention on the healthy lifestyle index which is consistent with reported literature advocating physical activity, weight management and healthy diet to optimize benefit of drug therapy on cardiovascular risk [22]. Previous studies have shown that GP practices offer an excellent avenue to address multiple risk factors including lifestyle modification [23]. Our study was designed to emphasize this important aspect of hypertension management during various points of contact with the individuals during and following the clinic visit. First, lifestyle advice was an integral part of physician treatment algorithm for all hypertensive individuals. Second, all individuals at high risk received MC by trained nurses. Use of MC is increasingly effective in the management of chronic diseases [24]. The MC curriculum used in our study was especially developed using local case-based scenarios, and focused on life style management and adherence to medications. Third, all individuals received monthly telephone follow-up during which a standardized checklist on lifestyle and medication adherence was administered by nurses trained in a MC, with counseling offered as needed. This strategy was also relatively low cost at S$ 4.5 for a monthly telephone call is an additional strength, although formal cost effectiveness needs to be evaluated in the full scale study. Finally, fixed-dose antihypertensive regimens offered to high risk individuals at a subsidized cost have been shown to improve adherence, as opposed to multiple single medications [25]. Nevertheless, our feasibility trial suggests that a packaged intervention using the unique combination of specified components is likely to improve the healthy life style score as well as adherence to antihypertensive medications in individuals seeking care in the busy polyclinics in Singapore.

The adjusted decline in systolic and diastolic BP within the intervention and the usual care groups was marked, however between-group differences were not significant. This suggests the possibility of a Hawthorne effect with BP regression to the mean in both groups at 3 months. However, the benefits of improved lifestyle and adherence to antihypertensive medications observed in the intervention group would require longer term follow up in a larger number of participants. An adequately powered study accounting for clustering is needed to study the effectiveness and cost effectiveness of the intervention on BP and other clinically meaningful outcomes. Our study underscores the need for a control group in these comparisons.

Finally, a limited participant satisfaction survey indicated that both providers and participants appreciated the utility of the strategies.

Our findings have limitations. The parallel groups were allocated clusters, and differences in outcomes do not account for clustered data.. However, the main aim of the feasibility study was to conduct a pilot of standardized protocol incorporating the main components of the intervention and using the polyclinic workforce infrastructure which we were able to achieve with high fidelity. Moreover, we were able to demonstrate acceptability of key intervention components in predominantly older adults—the fastest growing segment of Singapore population [26]. Thus, we believe a full scale study of the proposed strategies is feasible in the polyclinics to conclusively answer questions of clinical and cost effectiveness. The clues from our feasibility study on the healthy lifestyle index in conjunction to adherence to antihypertensive medications indicate success is likely for both aims.

## Conclusion

In conclusion, our study on 100 individuals with uncontrolled hypertension in two polyclinics in Singapore resulted in high intervention fidelity and signaled improvement in healthy life style factors and adherence to anti-hypertensive medications compared to usual care. The packaged intervention needs to be evaluated perhaps with additional measures to enhance FDC prescription in a full scale trial for effectiveness and cost effectiveness on BP lowering and improvement of cardiovascular risk for future scalability and sustainability. Our proposed trial has significant clinical practice and public health policy implications in Singapore, and globally.

## Abbreviations

ACEI, ACE inhibitors; ARB, angiotensin receptor blocker; BMI, body mass index; BP, blood pressure; CCB, calcium channel blockers; CME, continued medical education; CVD, cardiovascular diseases; DBP, diastolic blood pressure; FDC, fixed dose combination; GFR, glomerular filtration rate; HCTZ, hydrochlorothiazide; HLI, healthy lifestyle index; ICC, intra-cluster correlations; LOCF, last observation carried forward; MC, motivational conversation; MMAS-8, morisky medication adherence scale; NHS, national health surveys; PDC, Proportion of days covered; SBP, systolic blood pressure; SD, standard deviation.

## Additional files

Additional file 1: Checklist for Telephone follow-up by Nurse Practitioners and Nurses (table). (DOC 64 kb) Additional file 2: Healthy Lifestyle Index Score (table). (DOC 33 kb)
