# Supplementary figures and images for: Management of hypertension and multiple risk factors to enhance cardiovascular health - a feasibility study in Singapore polyclinics
Source: BMC Health Serv Res. 2016 Jul 8;16:229. doi: 10.1186/s12913-016-1491-6 (PMC4938988; doi:10.1186/s12913-016-1491-6)

**Additional file 1: Checklist for Telephone follow-up by Nurse Practitioners and Nurses**


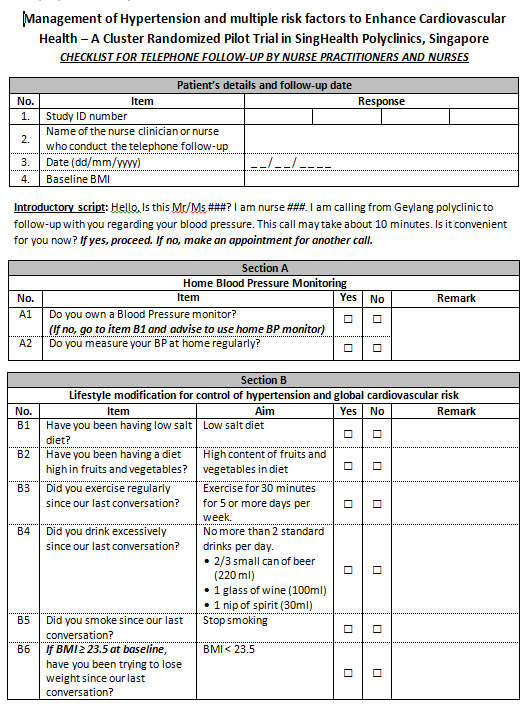

Supplement: Additional file 1: — Checklist for Telephone follow-up by Nurse Practitioners and Nurses (table). (DOC 64 kb) [file 12913_2016_1491_MOESM1_ESM.doc]
