# Supplementary material for: Management of hypertension and multiple risk factors to enhance cardiovascular health - a feasibility study in Singapore polyclinics
Source: BMC Health Serv Res. 2016 Jul 8;16:229. doi: 10.1186/s12913-016-1491-6 (PMC4938988; doi:10.1186/s12913-016-1491-6)
Supplement: Additional file 2: — Healthy Lifestyle Index Score (table). (DOC 33 kb) [file 12913_2016_1491_MOESM2_ESM.doc]

| **Additional file 2: Healthy Lifestyle Index Score** | | |
| --- | --- | --- |
| **Healthy Lifestyle Factors*^1,2,3,4,5^** | **Score-0** | **Score-1** |
| **BMI^2^** | ≥ 23.5 kg/m^2^ | <23.5 kg/m^2^ |
| **Physical Activity^3^** | <150min/week moderate and <75min/week vigorous-intensity | ≥150 min/week moderate or  ≥75min/week vigorous-intensity |
| **Dietary habit** | Dining at Hawker center | Never Dining at Hawker center |
| **Dietary quality** | Eating uncooked vegetables  < 4 times/month and  Eating fruits < 4 times/week | Eating uncooked vegetables  ≥ 4 times/month or  Eating fruits ≥ 4 times/week |
| **Smoking^4^** | Smoker | Non-smoker |

* Healthy Lifestyle Index; 0 (poor) ---> 5 (Excellent)

References:

1. Spring, Bonnie, et al. "Healthy lifestyle change and subclinical atherosclerosis in young adults: Coronary Artery Risk Development in Young Adults (CARDIA) study." Circulation (2014): CIRCULATIONAHA-113.
2. Lewis, Cora E., et al. "Mortality, Health Outcomes, and Body Mass Index in the Overweight Range A Science Advisory From the American Heart Association." Circulation 119.25 (2009): 3263-3271.
3. Ahmed, Haitham M., et al. "Low-risk lifestyle, coronary calcium, cardiovascular events, and mortality: results from MESA." American journal of epidemiology 178.1 (2013): 12-21.
4. Jiao, Li, et al. "A combined healthy lifestyle score and risk of pancreatic cancer in a large cohort study." Archives of internal medicine 169.8 (2009): 764-770.
5. <https://www.healthylifestyleindex.sg/asset/image/v2/hli_White_Paper_Summary.pdf>
